# The impact of warm ischemia time on short-term renal function after partial nephrectomy: a systematic review and meta-analysis

**DOI:** 10.1186/s12894-025-01803-w

**Published:** 2025-05-13

**Authors:** Siyi Li, Zhenlang Guo, Yuan Li, Franky Leung Chan, Shusheng Wang, Chiming Gu

**Affiliations:** 1 Department of Urology, The Second Affiliated Hospital of Guangzhou University of Chinese Medicine, Guangzhou, China; 2 School of Biomedical Sciences, The Chinese University of Hong Kong, Shatin, Hong Kong China

**Keywords:** Ischemia, Partial nephrectomy, Renal function, Systematic review, Meta-analysis

## Abstract

**Purpose:**

This study aimed to assess the impact of warm ischemia time on short-term renal function in individuals undergoing partial nephrectomy.

**Methods:**

We conducted a comprehensive search for primary research articles from 1990 to October 15, 2024 across several databases, including MEDLINE, Embase and the Cochrane Library. A random effects model was applied to determine multivariable adjusted odds ratios (ORs) and their corresponding 95% confidence intervals (CIs). Country and study design were utilised as outcome indicators in the regression model.

**Results:**

Ten studies including 4,993 patients who underwent partial nephrectomy met the inclusion criteria. The threshold of potentially harmful ischemia time for renal artery occlusion ranges between 10 and 45 min. Our results revealed that long warm ischemia time was associated with decreased postoperative eGFR and poor short-term renal function (OR = 1.08; 95% CI = 1.02–1.15; *P* = 0.006) after partial nephrectomy. Sensitivity and meta-regression analyses demonstrated the robustness of the study’s findings.

**Conclusions:**

Extended periods of warm ischemia, specifically exceeding 25–30 min, can inflict damage on kidneys undergoing surgical treatment. Minimising the duration of warm ischemia while simultaneously prioritising surgical safety and achieving clear margins is imperative. Moreover, ischemia time remains a modifiable risk factor and must be reduced to maintain overall short-term renal function. Relevant prospective and randomised controlled trials must be conducted to validate these findings.

**PROSPERO Registration number:**

CRD42024560051.

## Introduction

Current guidelines recommend partial nephrectomy (PN) as the optimal treatment for cT1 renal tumours, provided it is technically and oncologically viable [1, 2]. With the evolution of surgical methods, the application of PN has notably increased over the years. Current guidelines also endorse PN for patients with large tumours (classified as cT2), particularly in situations involving a solitary kidney or chronic kidney disease (CKD), provided the procedure is technically achievable [1, 2]. The foremost aim of PN is to effectively manage cancer by ensuring the complete removal of the tumour with sufficient margins [3]. Preserving renal function to the greatest extent is its secondary crucial goal. Extensive research has been conducted on different selective clamping and no-clamp techniques for PN [4, 5]. This focus stems from the contribution of renal ischemia to renal function deterioration after PN [6, 7]. Nonetheless, opting to perform renal surgery without clamping can elevate the likelihood of intra-operative complications, such as excessive bleeding, particularly in intricate cases [8]. This approach can complicate the surgical procedure and potentially lead to adverse effects on surgical and oncological outcomes, including a high incidence of positive surgical margins [9]..

Urologists have an ongoing discussion about how permissible warm ischemia time influences short-term renal function following PN. Hence, we conducted a comprehensive review and meta-analysis of existing literature to evaluate the effects of warm ischemia time on short-term renal function following PN.

## Materials and methods

This meta-analysis of original clinical studies was conducted in line with the PRISMA guidelines [10]. Hence, no ethical approval or informed consent from patients was necessary.

### Literature search

Relevant studies on the correlation between warm ischemia time and short-term renal function in individuals undergoing PN were sourced from several databases, including Embase, MEDLINE and the Cochrane Library, from 1990 to October 15, 2024. The database search encompassed all languages, publication types and regions. The search strategy included a mix of Medical Subject Headings (MeSH) and non-MeSH terms, detailed as follows: (‘nephrectomy’ OR ‘nephrectomies’ OR ‘partial nephrectomy’) AND (‘ischemia’). The bibliographies of past reviews and related articles were also manually reviewed to locate additional pertinent reports. Any discrepancies were resolved through agreement with the co-investigators.

### Inclusion and exclusion criteria

Firstly, two reviewers separately examined the titles and summaries of the initial studies to eliminate any that failed to meet the specified criteria, documenting the reasons for their exclusion. A detailed review of the full texts of potentially eligible studies was then performed. Studies that fulfilled the following eligibility criteria were included: (1) original research regarding the correlation between warm ischemia time and short-term renal function in individuals undergoing PN (i.e. open, laparoscopic or robot-assisted); (2) studies that included risk estimates (such as OR, hazard ratio [HR] and relative risk) with their 95% confidence intervals (CIs); if these were not provided, adequate raw data for calculation were necessary; and (3) clinical research (observational studies, randomised controlled trials, or cross-sectional studies). Serum creatinine, estimated glomerular filtration rate (eGFR), effective renal plasma flow from renal scintigraphy and split renal function of the treated kidney were evaluated prior to the procedure and at various intervals post-surgery. A decline in renal function exceeding 20%—calculated by eGFR at discharge compared to preoperative levels—was considered clinically significant. The primary objective was to evaluate the impact of warm ischemia time on postoperative short-term renal function, as measured by eGFR at discharge. For the evaluation of renal function at discharge, the final blood test conducted immediately prior to discharge was utilised as a surrogate for the most stable postoperative renal function attained. When duplicate publications were encountered, only the latest or highest-quality study was considered. Exclusion criteria included non-English articles, case reports, letters, reviews, and conference abstracts to ensure methodological consistency and focus on primary clinical studies. Any conflicts were resolved through consensus among the co-investigators.

### Data extraction and methodological quality evaluation

Two researchers independently gathered crucial data from the selected studies. The extracted details were organised into a standardised Excel file, including the first author, design, country, type of surgery and ischemia, sample size, tumour size, baseline eGFR, median ischemia time, potentially harmful ischemia time threshold, participant characteristics (such as average age), risk estimates with 95% CIs and confounders. If any studies lacked sufficient information, the lead author was contacted to obtain the missing data. Any disagreements during data extraction were resolved through consensus with the co-investigators.

The quality of the included studies was independently evaluated by two authors using the Newcastle–Ottawa Scale (NOS) [11], which includes 10 criteria. Each criterion was rated as ‘yes’ (scoring 1) or ‘no/unclear’ (scoring 0) based on the extracted study information. The overall score, ranging from 0 to 10, classified the studies as follows: scores of 8–10 indicated high quality, 5–7 indicated moderate quality and below 5 indicated low quality. Any disagreements were resolved through discussion among the co-researchers.

### Data integration and analysis

**Fig. 1:**
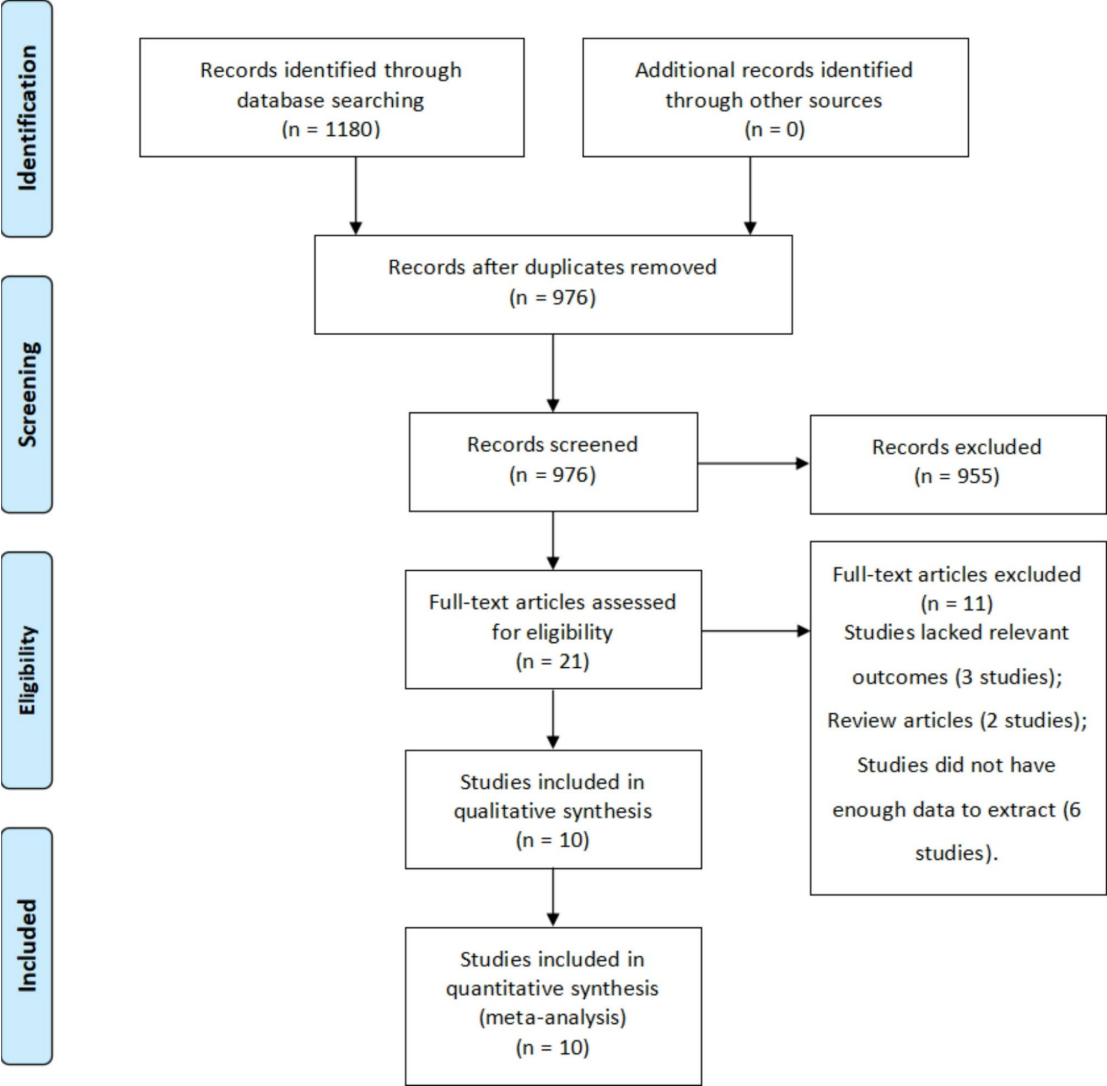
A flowchart of the literature search process following the guidelines of the PRISMA statement

Total risk estimate for each study was calculated using ORs and associated 95% CIs with STATA statistical software (version 15.0; serial number: 10699393; StataCorp Wyb). A flowchart (Fig. 1) generated from the PRISMA Flow Diagram shows the process of searching and screening literature [10]. Forest plots illustrate the aggregate findings and variability across studies. An I [2] test was conducted to assess the influence of variability on the meta-analysis results. Various I-values indicated varying levels of heterogeneity. In accordance with Cochrane review guidelines [12], a random effects model was applied when heterogeneity was I^2^ ≥ 50%; otherwise, a fixed-effects model was used. A threshold of *P* < 0.05 was established to determine statistical significance. Subgroup analyses by country and study design were performed to investigate how different methodologies and patient characteristics influence heterogeneity. Additionally, a sensitivity analysis was performed by excluding each study one at a time to test the robustness and consistency of the results. Meta-regression analyses using the restricted maximum likelihood method were carried out to identify potential sources of heterogeneity across various variables. The log odds ratio was set as the independent variable. Covariates included the study design and country, which are factors that may influence the results. The *p*-value was considered as the outcome indicator for the regression analysis to evaluate the statistical significance of this difference. Finally, Egger’s test was employed to assess publication bias [13]..

## Results

### Study identification and selection

Our initial search yielded 1180 records. After the duplicates were removed, 976 studies remained. Following a title and abstract screening, 955 studies were excluded, leaving 21 articles for full-text evaluation. Another 11 articles were excluded for the following reasons: three studies lacked relevant outcomes, two were review articles and six did not have enough data to be extracted (Fig. 1). Eventually, 10 original studies [14, 15, 16, 17, 18, 19, 20, 21, 22, 23] comprising 4,993 patients who underwent PN were included in this meta-analysis in accordance with the inclusion criteria.

### Study characteristics

Table 1 outlines the fundamental characteristics of the included studies. All of these studies were published in English, with five being retrospective [14, 18, 19, 20, 22], four being prospective [16, 17, 21, 23] and one being a randomised controlled trial [15], between 1980 and 2021. The sample sizes varied from 44 patients to 1,816 patients. The reported median ischemia time during PN ranged from 10.5 min to 57 min. Except for one study [18], the threshold for potentially harmful ischemia time was reported in all studies and ranged from 10 min to 45 min. The mean tumour size ranged from 2.4 cm to 4 cm. Three of the studies were conducted in Italy [15, 17, 21], four in America [16, 18, 19, 22], two in Korea [20, 23] and one in Japan [14]. Each included study provided risk estimates that were adjusted for confounding variables.

**Table 1:** Characteristics of the included studies

Study	Design	Country	Type of surgery	Sample size	Mean tumor size (cm)	Baseline eGFR, mL/min/1.73 m2, mean (range)	Mean age (y)	Median ischemia time (min)	Potentially harmful ischemia time threshold (min)
Abe T et al. 2012 [14]	Retrospective study (2003–2010)	Japan	LPN	61	2.4 (1-4.1)	79.0 (54.1-114.5)	60 (20–81)	57 (34–112)	45
Antonelli AD et al. 2023 [15]	Randomized controlled trial (2015–2018)	Italy	LPN	324	3 (2–4)	87.12 (75.91–96.52)	65 (55–71)	10.5 (0–16)	10
Beksac AT et al. 2020 [16]	Prospective study (2006–2018)	America	RAPN	375	3.2 (2.4–4.4)	50.5 (42.9–56.3)	68 (61–74)	NA	15
Cignoli D et al. 2023 [17]	Prospective study (1988–2021)	Italy	LPN	1140	3 (2.4-4.0)	85.6 (67.1–98)	61 (51–70)	15 (5.8–20)	15
Thompson RH et al. 2010 [18]	Retrospective study (2000–2009)	America	LPN and OP	458	NA	NA	NA	NA	NA
Lane BR et al. 2010 [19]	Retrospective study (1980–2008)	America	LPN	360	4 (2.8–5.3)	51 (41–64)	63 (54–70)	32 (21–47)	30
Lee H et al. 2018 [20]	Retrospective study (1998–2016)	Korea	LPN	1,816	2.4	NA	54.6	NA	30
Porpiglia F et al. 2012 [21]	Prospective study (2006–2008)	Italy	LPN	53	3 (1.6)	NA	58.6 (13.2)	21.9 (8.6)	30
Thompson RH et al. 2012 [22]	Retrospective study (1990–2008)	America	LPN and OP	362	3.4 (0.7–18)	NA	62 (19–93)	21 (4–55)	25
Choi JD et al. 2010 [23]	Prospective study (2008–2009)	Korea	LPN and RAPN	44	2.5 (0.8–5.7)	89.4 (37.4–133)	53.5 (21–81)	33.4 (19–58)	28

Note: OP, open partial nephrectomy; LPN, laparoscopic partial nephrectomy; RAPN, robot-assisted partial nephrectomy; GFR, glomerular filtration rate; NA, not available

### Methodological quality assessment

The methodological quality of the included studies was assessed using the NOS. Three studies [14, 15, 17] scored 9 or 10, indicating high quality. Six studies [16, 19, 20, 21, 22, 23] received 7 or 8 points, classifying them as moderate quality. The remaining study [18] scored 5 points and was deemed to be of low quality.

### Impact of warm ischemia time on short-term renal function after PN

**Fig. 2:**
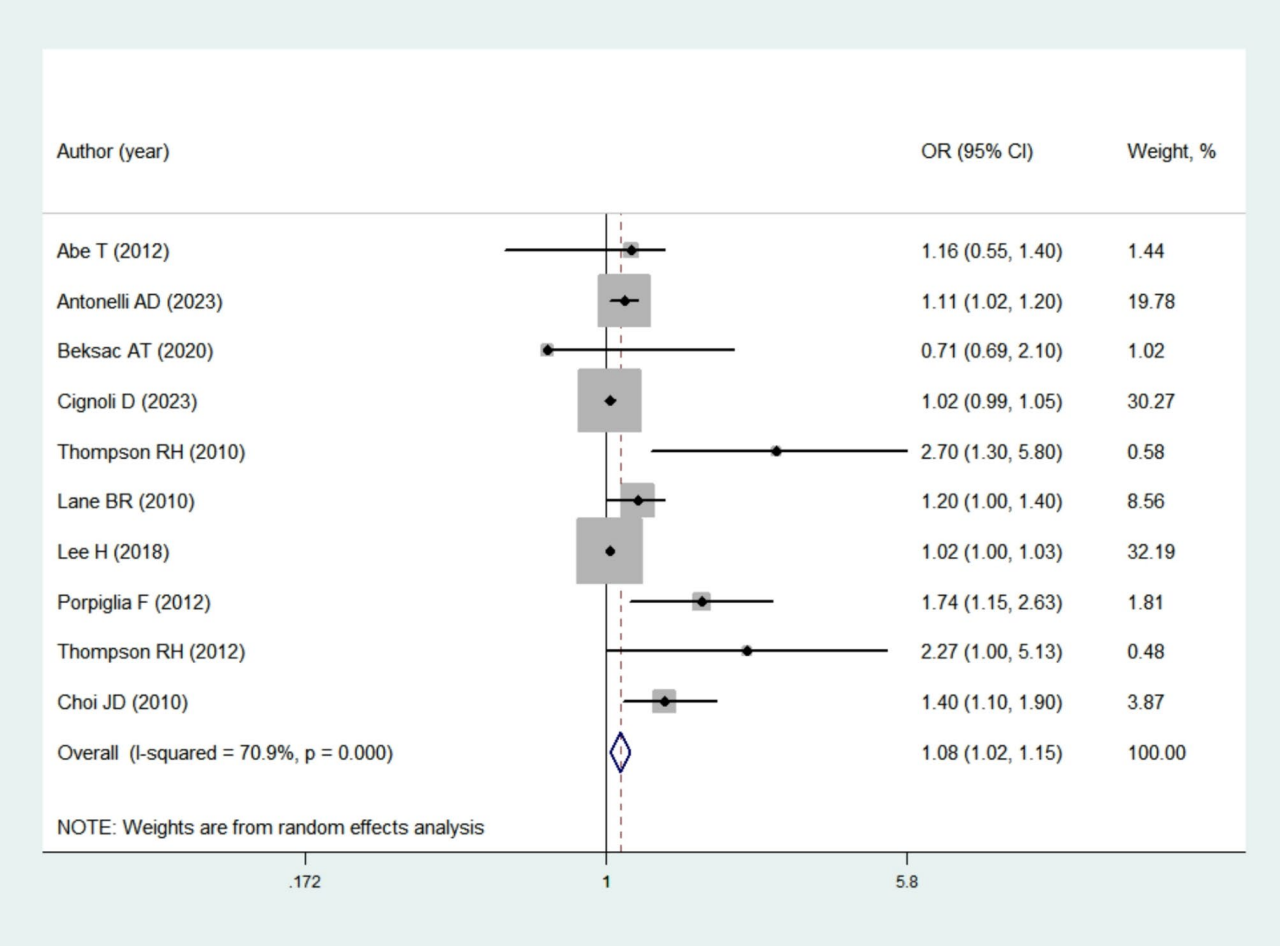
The impact of warm ischemia time on renal function after partial nephrectomy. CI, confidence interval, OR, Odds Ratio

Ten studies [14, 15, 16, 17, 18, 19, 20, 21, 22, 23] offered adequate data on the relationship between warm ischemia time and short-term renal function in individuals undergoing PN. After the adjustment for confounding variables, the findings indicated that long warm ischemia time was associated with decreased postoperative eGFR and poor short-term renal function (OR = 1.08; 95% CI = 1.02–1.15; *P* = 0.006) after PN. Owing to the significant heterogeneity, a random effects model was employed for the pooled analysis as depicted in Fig. 2. Our subgroup analyses revealed that when the studies were stratified by different countries (Table 2), the pooled effect size was not statistically significant which may be due to the limited number of studies. When the subgroups were divided by different study designs, significant results were observed in the randomised controlled studies (OR = 1.11; 95% CI = 1.02–1.20; *P* = 0.012). By contrast, the retrospective studies (OR = 1.23; 95% CI = 0.99–1.53; *P* = 0.062) and prospective studies (OR = 1.18; 95% CI = 0.88–1.57; *P* = 0.270) yielded non-significant findings. Significant results were found regardless of whether the threshold for potentially harmful ischemia time was 10–15 min (OR = 2.80; 95% CI = 2.72–2.88; *P* < 0.05) or 25–30 min (OR = 2.78; 95% CI = 2.74–2.82; *P* < 0.05).

**Table 2:** Results of subgroup analyses

Overall results	Studies, *N*	OR (95% CI)	*p* value	I ^2^ (%)
	10	1.08 (1.02–1.15)	0.006	70.9
Study design				
Prospective studies	4	1.18 (0.88–1.57)	0.270	77
Randomized controlled trials	1	1.11 (1.02–1.20)	0.012	NA
Retrospective studies	5	1.03 (1.00–1.06)	0.062	71.4
Country				
Japan	1	1.16 (0.73–1.85)	0.533	NA
Italy	3	1.10 (0.97–1.24)	0.125	79.6
America	4	1.39 (0.85–2.26)	0.186	70.7
Korea	2	1.16 (0.86–1.57)	0.342	80.6
Ischemia time				
10–15 min	3	2.80 (2.72–2.88)	< 0.001	52.9
25–45 min	5	2.78 (2.74–2.82)	< 0.001	65.4

Note: CI, confidence interval, OR, Odds Ratio; NA, not available

**Fig. 3:**
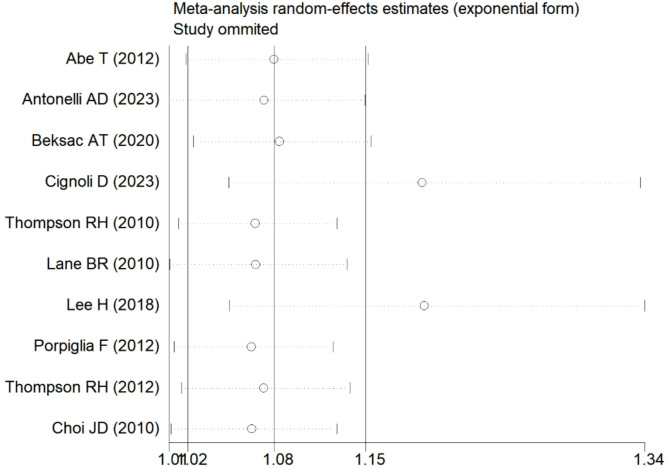
Results of sensitivity analyses

**Fig. 4:**
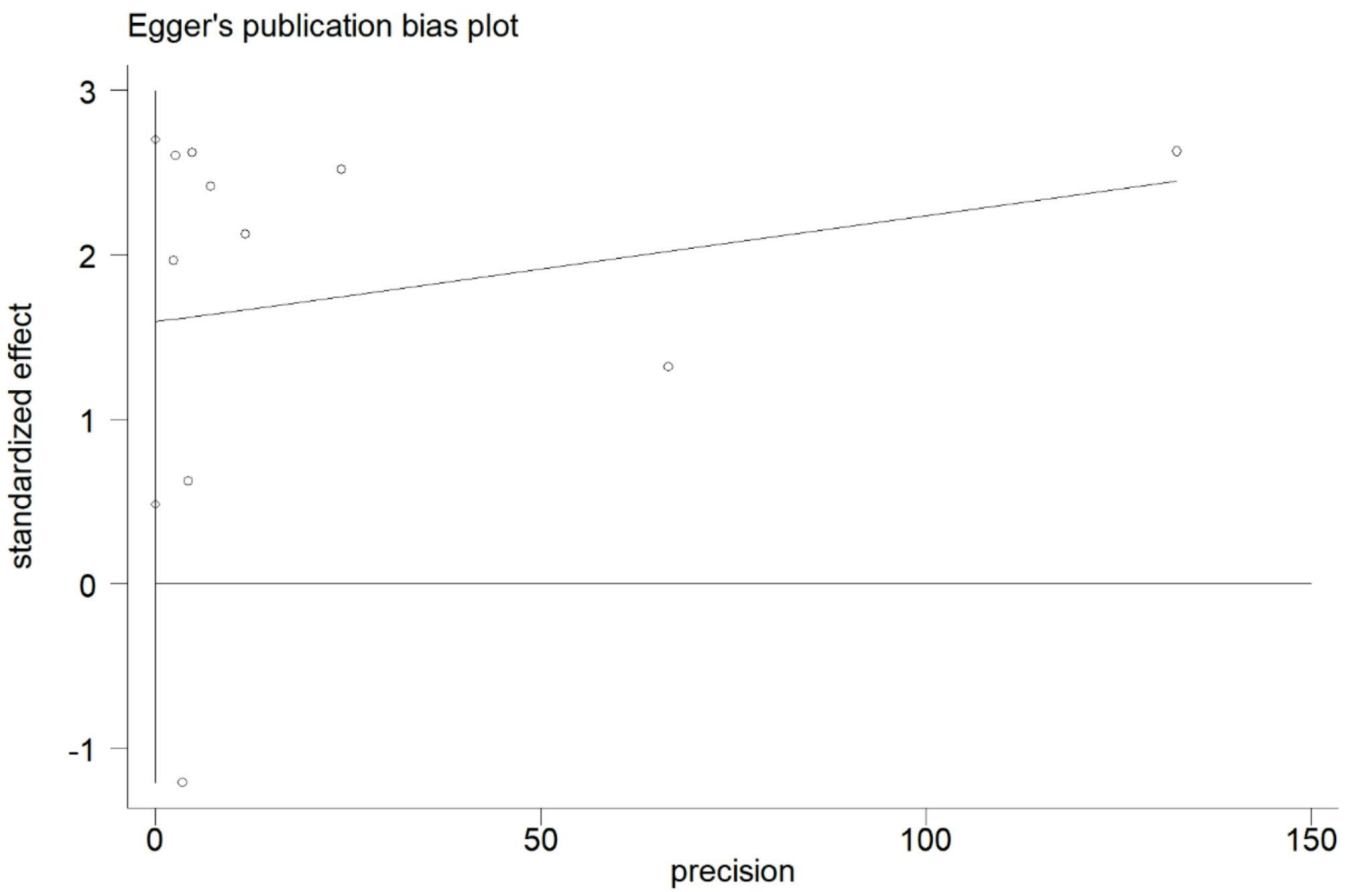
Results of Egger’s test with funnel plot

Sensitivity analyses revealed that excluding each study individually did not significantly alter the overall results (Fig. 3). A meta-regression analysis was conducted to further investigate the observed heterogeneity among the studies. The results indicated that none of the covariates, including country (*P* = 0.998) and study design (*P* = 0.691), contributed to the heterogeneity. The adjusted R-squared value of − 233.73% suggested that the regression variables had a negligible impact on explaining the response variables. Lastly, Egger’s test results showed no evidence of publication bias (Egger’s test, *P* = 0.538) (Fig. 4).

## Discussion

### Main findings

This meta-analysis investigated the link between warm ischemia time and short-term renal function in individuals undergoing PN. Results indicated that extended periods of warm ischemia, specifically exceeding 25–30 min, can inflict irreversible damage on kidneys undergoing surgical treatment. Sensitivity analyses showed that the overall stability of the findings remained consistent despite the exclusion of individual studies. Although the meta-regression failed to pinpoint specific factors contributing to inter-study heterogeneity, no evidence of publication bias was detected based on Egger’s test and funnel plot analysis.

Two of the studies reported negative results regarding this issue [16, 17]. Beksac et al. [16]. conducted a prospective study on 375 patients who underwent PN and found no significant difference in eGFR change between the two groups when the ischemia time threshold was 15 min. Cignoli et al. [17]. found that extended warm ischemia time was not correlated with a reduction in postoperative eGFR in multivariable analyses. Additionally, no link was observed between warm ischemia time and post-eGFR at either the 6-month mark or during long-term monitoring. Patients and healthcare providers must recognise that executing PN with minimal or no warm ischemia time may lead to high risks of bleeding and a great likelihood of requiring peri-operative blood transfusions, without yielding good long-term renal function results. Additional studies on this issue must be published in the future to guide clinical practice.

### Implications for clinical practice

Over the past decade, extensive research has focused on the connection between warm ischemia time and short-term renal function following PN. The impact of warm ischemia time and ischemia type on the deterioration of renal function post-surgery remains contentious [24]. Most researchers adhere to a traditional limit of 25–35 min [25]. However, histopathological studies have challenged this boundary, demonstrating that the human kidney can endure extended periods of warm ischemia [26, 27]. A further complication in thoroughly examining postoperative functionality involves evaluating the impact on the same side and renal function while considering the compensatory mechanisms of the opposite side. Only a limited number of retrospective studies have specifically explored the functional outcomes of PN through renal scintigraphy. These investigations typically depend on small sample sizes or subgroups derived from large datasets where scintigraphy is not routinely recommended, leading to potential selection bias [28, 29]. Moreover, three investigations analysed the effects of cold versus warm ischemia on renal function after surgery. La Rochelle et al. [30]. observed comparable reductions in renal function between 22 patients subjected to brief warm ischemia (average 12 min) and 12 patients experiencing an average of 33 min of cold ischemia. A similar comparison was made between 300 partial nephrectomies conducted on solitary kidneys under cold ischemia and 360 surgeries performed under warm ischemia, with adjustments for parenchymal preservation. The researchers found no significant difference in renal function preservation between the two groups in univariate and multivariable analyses [19]. Two strategies have been proposed to enhance the retention of parenchymal mass during PN, reduce the amount of parenchyma removed and avoid the loss of blood supply to nearby parenchyma during suturing [31]. Best clinical practices must be followed, specifically, maintaining short warm ischemia times and using hypothermia as needed. Therefore, ischemia duration is not the primary determinant of long-term postoperative renal function. Instead, the extent and quality of preserved renal parenchyma play a more crucial role. Although consistent data linking intraoperative ischemia to end-stage renal disease in both kidneys are lacking, warm ischemia time remains a significant predictor of acute renal failure [32]. Therefore, maintaining the warm ischemia time below this threshold is essential and potentially advantageous. Earlier research evaluated the Acute Dialysis Quality Initiative’s acute kidney injury (AKI) classifications as indicators of long-term kidney failure following PN. The Risk, Injury, Failure, Loss, and End-Stage criteria characterise AKI as a sudden decline in kidney function, marked by a more than 25% decrease in baseline eGFR, which is similar to our results [33, 34]. AKI heightens the likelihood of mortality and CKD in patients with pre-existing health issues, which is previously believed to not influence outcomes in patients undergoing PN [35, 36]. However, recent investigations have revealed that the occurrence and duration of AKI significantly elevate the risk of long-term renal failure in these individuals [37]. Although the relationship between AKI occurrence after PN and long-term kidney function impairment is important, the data on AKI in the original included literature is quite limited, which restricts our understanding in this area. We hope that future research will focus on this topic. The selection of eGFR at discharge as the primary outcome warrants further consideration. Postoperative AKI often leads to transient creatinine elevation, peaking 2–3 days post-surgery, followed by gradual recovery [34, 37]. While discharge eGFR represents a standardized time point for comparison across studies, it may reflect an unstable phase of renal function recovery. For instance, creatinine levels typically stabilize weeks after surgery, and long-term eGFR measurements (e.g., at 3 months) might better reflect sustained renal outcomes. However, heterogeneity in follow-up timing among included studies (e.g., limited availability of 3-month eGFR data) constrained our ability to assess long-term trajectories. Future studies should prioritize serial eGFR measurements to delineate the dynamic recovery process and validate discharge eGFR as a reliable surrogate. While minimizing warm ischemia time is paramount to preserving renal function, surgeons must balance this objective with adherence to oncological principles and intraoperative safety. Positive surgical margins (PSMs) remain a key determinant of cancer-specific survival [38], and overly aggressive efforts to reduce ischemia time may compromise tumor resection completeness. For instance, hurried parenchymal dissection under time constraints risks leaving residual tumor, particularly in complex or endophytic lesions [39]. Conversely, meticulous tumor excision to ensure negative margins may necessitate prolonged ischemia, highlighting a delicate trade-off between oncological efficacy and functional preservation. Similarly, hemorrhage control directly impacts warm ischemia time management. Excessive bleeding during unclamped or early-unclamping techniques may force surgeons to reclamp the renal artery, inadvertently prolonging ischemia [40]. Studies by Mir et al. demonstrated that transfusion-requiring bleeding during PN independently correlates with prolonged warm ischemia time (*P* = 0.02) and acute kidney injury [31]. Thus, preoperative planning and selective use of advanced hemostatic agents are essential to optimize both ischemia duration and surgical safety. Emerging strategies, such as super-selective arterial clamping [41] or intraoperative indocyanine green fluorescence imaging [42], aim to reconcile these competing priorities. For example, Zhang et al. reported that super-selective clamping reduced median warm ischemia time by 8 min compared to main artery clamping (*P* < 0.001) without increasing PSMs or bleeding complications [41]. These innovations underscore the importance of individualized surgical approaches tailored to tumor anatomy and patient comorbidities.

Our study focused on elucidating the dose-dependent relationship between warm ischemia time and renal functional decline. However, non-clamping techniques, such as off-clamp PN or super-selective arterial embolization, offer an alternative strategy to eliminate ischemia entirely, particularly in anatomically favorable tumors [40]. While these approaches are not directly comparable to clamped cohorts in our meta-analysis (due to the absence of warm ischemia time data), they underscore the broader clinical imperative to minimize ischemic injury. Nevertheless, non-clamping techniques are not without limitations. Key complications include: (1) Increased Intraoperative Bleeding Risk: Off-clamp PN is associated with higher rates of significant hemorrhage (OR = 2.1, 95% CI: 1.4–3.0) [42], which may necessitate transfusion or conversion to clamping [41]. (2) Tumor Complexity Constraints: Non-clamping is often restricted to small, exophytic lesions (RENAL score ≤ 7) [40], limiting applicability in centrally located or large tumors. (3) Technical Demands: Superselective embolization requires advanced imaging and surgical expertise, potentially prolonging operative time [43]. Despite these challenges, non-clamping strategies hold promise for renal preservation. For example, Gill et al [40]. reported a 95% renal function retention rate at 6 months post-zero-ischemia PN. However, existing evidence is predominantly derived from single-center, retrospective series with limited sample sizes and short follow-up [37]. Large-scale, prospective studies are urgently needed to validate long-term oncological safety, refine patient selection criteria, and standardize technical protocols. Future research should also compare non-clamping outcomes with optimized ischemia protocols (e.g., warm ischemia time ≤ 20 min) to delineate the optimal balance between renal protection and surgical feasibility.

Moreover, clinicians should be aware that performing PN with very limited warm ischemia time may increase the risk of bleeding and the need for perioperative transfusions, without improving long-term renal function. These findings highlight the importance of other renal function determinants, such as the percentage of residual renal parenchyma. Therefore, we recommend that surgeons always prepare for renal pedicle clamping during PN and perform ischemic management immediately in case of bleeding to preserve as much renal parenchyma as possible. Unfortunately, most original literature databases do not allow the interpretation of renal function impairment markers, such as loss of renal parenchyma volume, even though all the studies adjusted for factors related to residual renal parenchyma volume, such as tumour size. This restriction significantly limits our ability to determine other factors that may influence short-term renal function. We hope that high-quality studies in the future will adjust for the percentage of residual renal parenchyma when focusing on WIT so that the results will be highly reliable.

### Strength and limitations

This meta-analysis offers several key advantages. Firstly, it is the first meta-analysis to investigate the correlation between warm ischemia time and short-term renal function in individuals undergoing PN, with subgroup analyses according to country, study design and ischemia time threshold to assess the impact of these variables on heterogeneity following PRISMA guidelines. Secondly, multivariable-adjusted risk estimates were employed to minimise the influence of confounding factors on the overall results. Lastly, the robustness and validity of our findings were confirmed through meta-regression and sensitivity analyses.

This meta-analysis also has several limitations. Firstly, some of the included studies are retrospective, which may introduce disadvantages such as missing data and potential bias. Secondly, incomplete retrieval due to non-extractable data is another concern. Thirdly, significant heterogeneity was observed. Although a meta-regression was applied, the risk of introducing substantial heterogeneity remained. Our objective in conducting meta-regression analysis is to evaluate the magnitude and sources of heterogeneity among the studies to identify factors that may influence the stability of the results. Owing to the limited number of the studies, other factors may have not been thoroughly investigated. We hope that future research will validate the stability and reliability of our findings. Lastly, all of the included studies reported short-term renal function at the time of discharge from the hospital. Conversely, significant association may be not observed between warm ischemia time and eGFR at the 6-month or long-term follow-up. Our understanding of the impact of warm ischemia time with long-term follow-up after PN is limited because these crucial details were inadequately reported in the included studies. Therefore, further high-quality research is warranted to determine other potential risk factors affecting renal function after PN.

## Conclusions

Our findings indicate that extended warm ischemia time more than 25–30 min may lead to ischemic damage in kidneys undergoing surgery. Therefore, keeping the warm ischemia time within this limit may be beneficial. Ischemia time remains a modifiable risk factor and should be minimized to preserve overall renal function, provided surgical safety and oncological efficacy are not compromised. Relevant prospective and randomised controlled trials must be conducted to confirm these findings.

## Data Availability

The data that support the findings of this study are available from the corresponding author, Chiming Gu, upon reasonable request.
